# Long-term results of the implantation of the AMS 800 artificial sphincter for post-prostatectomy incontinence: a single-center experience

**DOI:** 10.1590/S1677-5538.IBJU.2017.0165

**Published:** 2018

**Authors:** Carlos Alberto Ricetto Sacomani, Stênio de Cássio Zequi, Walter Henriques da Costa, Bruno Santos Benigno, Rodrigo Sousa Madeira Campos, Wilson Bachega, Gustavo Cardoso Guimarães

**Affiliations:** 1 A.C. Camargo Cancer Center-Fundação Antonio Prudente, São Paulo, SP, Brasil

**Keywords:** Urinary Incontinence, Urinary Sphincter, Artificial, Therapeutics

## Abstract

**Objectives:**

Report the long-term outcomes of the AMS 800 artificial sphincer (AS) for the treatment post-prostatectomy incontinence (PPI) in a single center in Brazil.

**Materials and Methods:**

Clinical data from patients who underwent the procedure were retrieved from the medical records of individuals with more than 1 year of follow-up from May 2001 to January 2016. Continence status (number of pads that was used), complications (erosion or extrusion, urethral atrophy, and infection), malfunctions, and need for secondary implantation were evaluated. The relationship between complications and prior or subsequent radiation therapy (RT) was also examined.

**Results:**

From May 2001 to January 2016, 121 consecutive patients underwent AS implantation for PPI at an oncological referral center in Brazil. At the last visit, the AS remained implanted in 106 patients (87.6%), who reported adequate continence status (maximum of 1 pad/day). Eight-two subjects (67.8%) claimed not to be using pads on a regular basis at the final visit (completely dry). Revision occurred in 24 patients (19.8%). Radiation therapy (RT) for prostate cancer following radical prostatectomy was used in 47 patients before or after AS placement. Twelve patients with a history of RT had urethral erosion compared with 3 men without RT (p=0.004).

**Conclusion:**

Considering our outcomes, we conclude that AS implantation yields satisfactory results for the treatment of PPI and should remain the standard procedure for these patients. Radiation therapy is a risk factor for complication.

## INTRODUCTION

Urinary incontinence (UI) can occur in 2% to 87% (1) of patients who undergo radical prostatectomy for prostate cancer. This variation reflects the disparate criteria that are used to define UI between studies, but most of the series have reported a 2% to 10% prevalence of post-prostatectomy incontinence (PPI) (2-5). Sphincter weakness is considered the main cause of PPI and can manifest alone or in association with bladder dysfunction (overactive detrusor and/or poor compliance) (6-9)). Implantation of the AMS 800 artificial sphincter (AS) is the standard procedure for treating PPI (10-13). However, other options, such as male slings, are becoming available (14-17).

The AS can be implanted using a perineal or scrotal approach, the results of which vary (18, 19).

Previous radiation therapy, concomitant urethral stenosis or bladder neck obstruction, and the experience of the surgeon can impact the outcomes following AS implantation. Erosion and extrusion are possible complications and occur four times more frequently with re-implanted devices compared with first-time insertion (20, 21). Urethral atrophy and malfunction are also well documented (18, 22). These issues should be addressed when an AS is offered to the patient.

Although implantation with an AS has been studied extensively, the Brazilian health agency only recently (2014) approved its coverage and reimbursement. Thus, Brazil will experience higher rates of AS implantation in the next several years. Since our institution has significant experience with the AS (since 2001), we decided to evaluate and report our results in the first long-term analysis at a Brazilian center.

## MATERIALS AND METHODS

This retrospective study analyzed the long-term outcomes of AS implantation for PPI at a single-center institution in Brazil. Clinical data from patients who underwent the procedure were retrieved from the medical records of individuals with more than one year of follow-up, from May 2001 to January 2016. Continence status (number of pads used), complications (erosion or extrusion, urethral atrophy, and infection), malfunctions, and need for secondary implantation were evaluated. The relationship between complications and prior or subsequent radiation therapy (RT) following AS implantation was also examined. The Clavien-Dindo system (23) was used to categorize the complications.

All procedures were conducted by three surgeons who were experienced with the technique.

Continence status was considered to be adequate when the patients reported using less than one pad per day and declared their satisfaction with the device. Men were considered to be completely dry when they claimed to have no regular need for pads (10-12).

Patients were also divided by access site (perineal or scrotal) and RT before or after AS placement.

**Figure 1:**
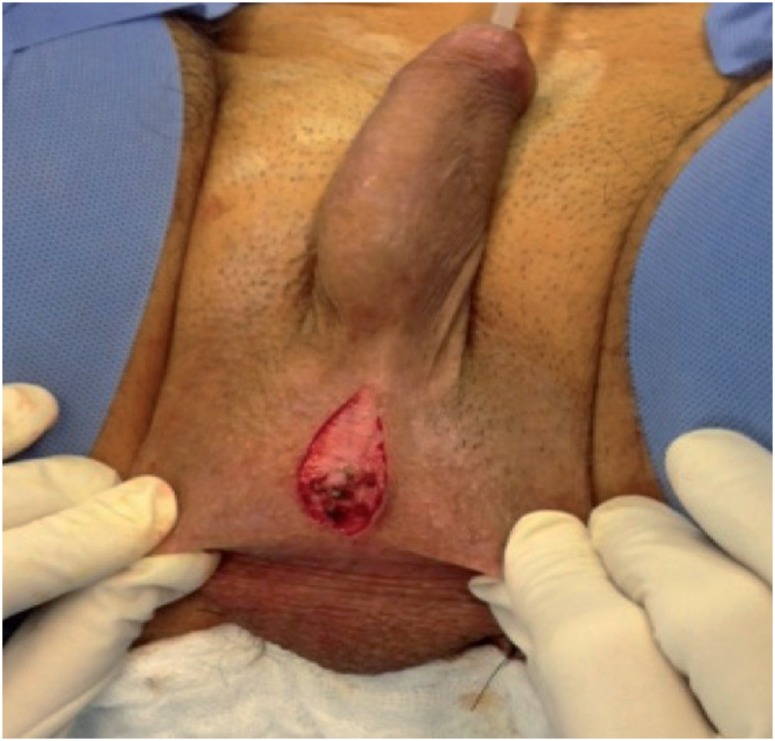
Longitudinal incision for modified scrotal approach.

**Figure 2:**
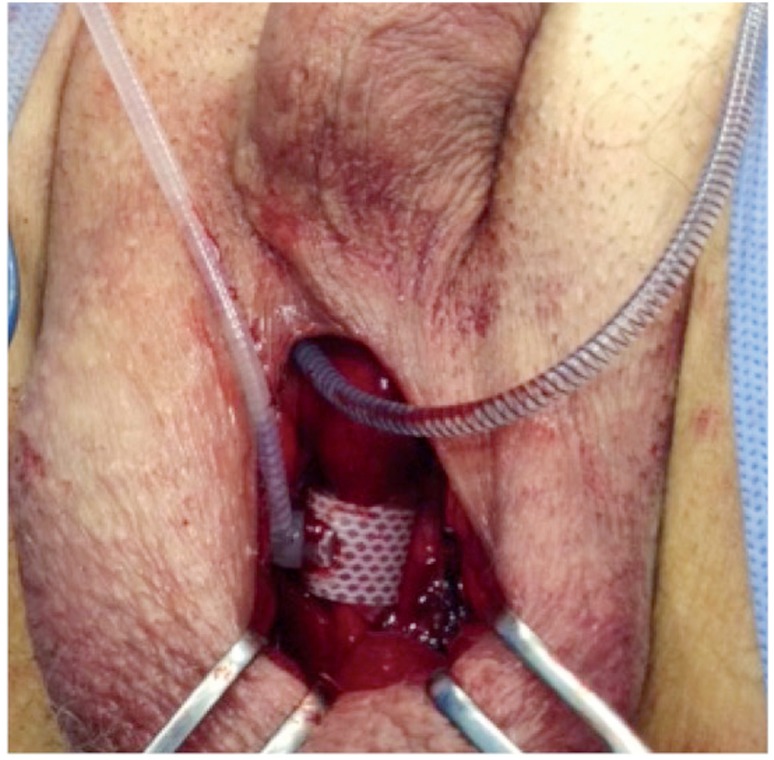
Bulbar urethra placement of As in modified scrotal approach.

For scrotal insertion of the AS, we used a modified technique, instead of that of Wilson et al. (19). We preferred a longitudinal incision and the cuff to be implanted toward the bulbous urethra. The regulator balloon was inserted through the superficial inguinal ring and placed above the fascia transversalis. The control pump and all connections were placed in the scrotum. Figures 1 and 2 show the scrotal incision and cuff placement.

Data were expressed as mean (or medians) ± standard deviation (minimum and maximum values) when applicable. IBM SPSS^®^ version 22 for Mac (SPSS Inc., Chicago, IL, USA) was used for the statistical analysis. Fisher's exact test was used to compare categorical data, with p<0.05 considered to be significant.

## RESULTS

From May 2001 to January 2016, 121 consecutive patients underwent AS implantation for PPI at an oncological referral center in Brazil. The mean follow-up was 5.2 years (range: 1.2 to 11.6 years; median 4.7 years). Seventy-one subjects received the AS through a perineal approach versus 50 by scrotal access. At the last visit, the AS remained implanted in 106 patients (87.6%), who reported adequate continence status (maximum of 1 pad/day). Eighty two subjects (67.8%) claimed not to be using pads on a regular basis at the final visit (completely dry). Revision occurred in 24 patients (19.8%) due to malfunction (1 case), urethral atrophy (5 cases), persistent UI (3 cases), and urethral erosion with or without skin extrusion (15 patients). Of the 106 patients with adequate continence, 9 patients underwent more than one procedure to attain the goal of using less than one pad/day.

In 11 patients, a tandem cuff (double cuff) was implanted. Four individuals received a second cuff due to urethral atrophy, compared with 3 for persistent UI and 4 due to severe incontinence (continuous leakage with no possibility of any bladder filling) at the first evaluation. Trans-corporeal placement was used in 7 cases (4 as salvage technique for the re-implantation following previous erosion and 3 as the first choice due to previous radiation therapy and multiple visual urethrotomies).

Urethral erosion (Clavien-Dindo III) occurred in 15 patients (12.4%), and skin extrusion (Clavien-Dindo III) appeared in 9 (7.4%). All patients with skin extrusion also developed urethral erosion. Two patients with a double cuff had urethral erosion and are included in the rates of this complication above. In one of the patients, erosion occurred after insertion of an indwelling catheter without deactivation of the AMS 800 during a heart attack at another institution.

**Figure 3:**
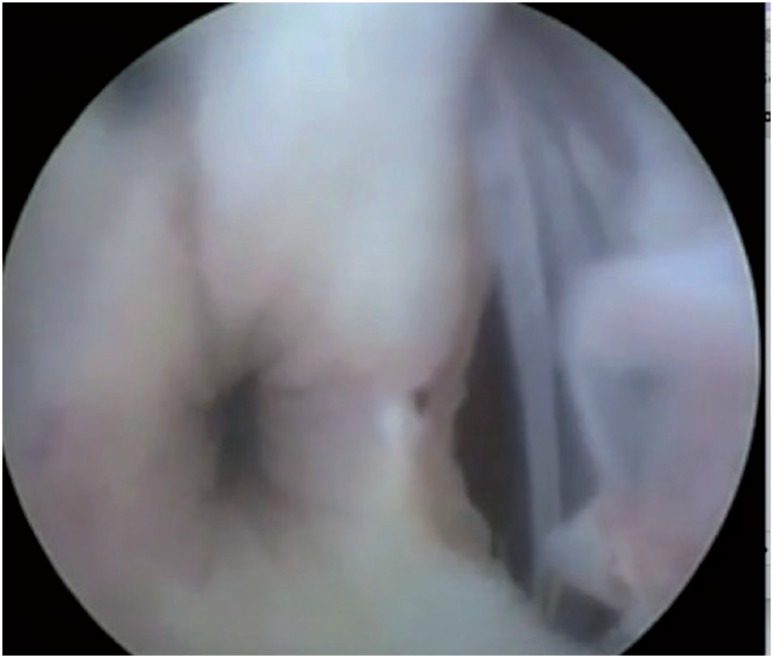
Urethral erosion of As. View from urethrocistoscopy.

**Figure 4:**
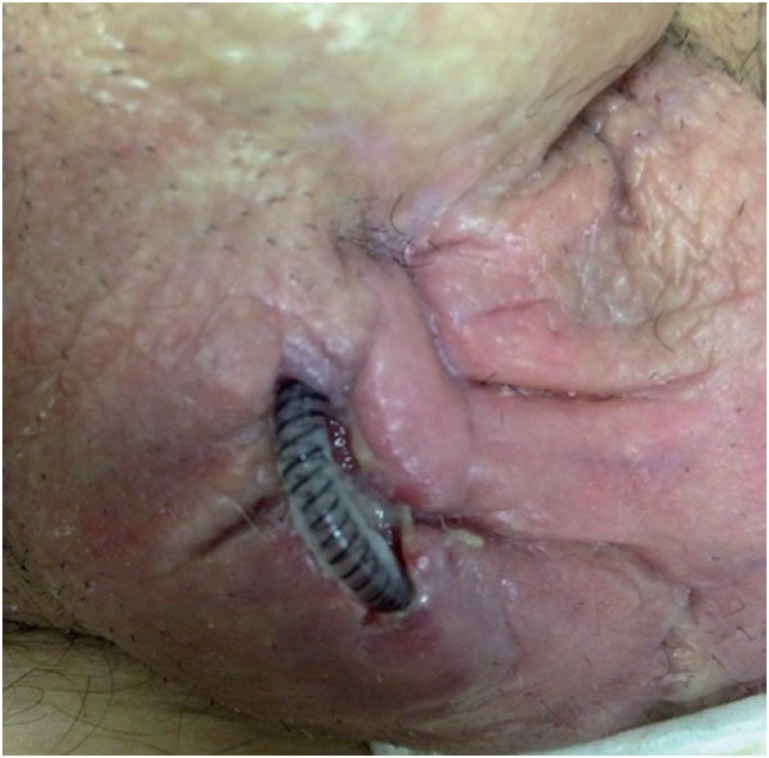
Skin extrusion of the As tube connecting the control pump to the regulator balloon.

**Figure 5:**
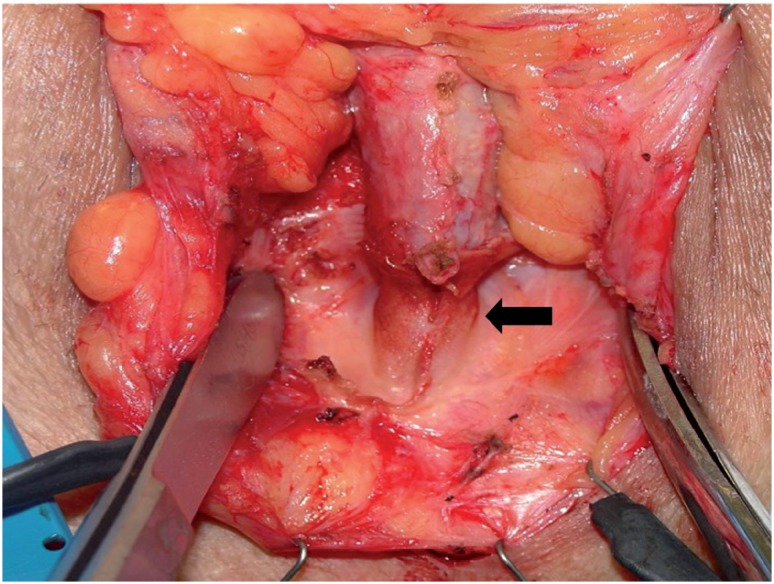
Urethral atrophy in a patient with previous AS placement.

Figures 3 and 4 show urethral erosion and skin extrusion, respectively. Urethral atrophy appears in Figure-5.

Table-1 summarizes the outcomes of AS placement at our institution.

**Table 1:** Outcomes of As placement at our institution from May 2001 to January 2016.

Outcomes	n (%)
Adequate continence	106 (87.6)
Completely dry	82 (67.8)
Urethral erosion/skin extrusion	15 (12.4)
Urethral atrophy	5 (4.1)
Malfunctioning	1 (0.8)
Persistent I.U. after first procedure	3 (2.5)
Revision rate	24 (19.8)

Nine patients (18.0%) who were submitted to AS implantation using a scrotal approach developed urethral erosion versus 6 (8.4%) after the perineal technique (p=0.16).

Radiation therapy (RT) for prostate cancer following radical prostatectomy was used in 47 patients before or after AS placement. Twelve patients with a history of RT had urethral erosion compared with 3 men without RT (p=0.004).

Table-2 shows the number of erosions in irradiated and non-irradiated patients.

**Table 2:** Urethral erosion after As placement at our Institution from May 2001 to January 2016 in Irradiated and non-Irradiated patients.

Groups	n (%)	Erosion n (%)
Irradiated patients	47 (38.9)	12 (25.5)
Non-irradiated patients	74 (61.1)	3 (4.0)

p=0.004

## DISCUSSION

The AS was developed by Brantley-Scott in 1983 (24) and remains the standard tool for treating PPI. Despite the good results with this device, there are controversies regarding its complications and survival. A recent study by the Mayo Clinic retrospectively analyzed 1082 implantations at that institution, showing that device survival declined continuously from 74% at 5 years to 41% at 15 years (11). However, the mean follow-up was 4.1 years, and only 60 devices were available for evaluation at 15 years.

Our study analyzed patients over 15 years of experience with AS placement at a Brazilian center. The mean follow-up was 5.2 years. At the final visit, 87.6% of patients had adequate continence. However, we could not determine whether there was a decrease in continence status due to the lack of a more objective analysis, such as the pad test and a quality of life questionnaire. The medical records only reported the number of pads that was used by the patient and a subjective evaluation of satisfaction.

The patient desires to be completely dry (especially with an expensive device implanted), but the AS might fail to provide total continence in all subjects. Eighty-two men claimed that they used pads only on certain occasions, such as intense physical activity. These individuals also described leakage eventually during coughing and sneezing, but these instances were insufficient to consider the use of a pad daily. To improve results, Kowalczyk et al. (25) described the insertion of 2 cuffs (a double cuff) in separate areas of the bulbous urethra. However, most urologists prefer to use the double cuff in select cases. In our study, 11 patients received two cuffs, primarily due to urethral atrophy or persistent IU. At the outset of our experience, we treated 4 patients with a double cuff due to continuous leakage, but this practice has been discontinued.

Erosion and extrusion are devastating complications after AS placement. The risk of erosion is four times higher with re-implanted devices compared with primary implantation. Urethral erosion and device extrusion often occur simultaneously. In our series, 12.4% of patients developed these complications. Compared with other studies, we noted more instances of erosion and extrusion. Previous radiation therapy (RT) could be related to the incidence of such outcomes. From 15 individuals with urethral erosion and extrusion, 12 had prior RT. Ravier et al. (26) also described worse results in a small cohort of irradiated patients. However, in a large series, Rivera et al. (27) reported similar outcomes in irradiated and non-irradiated individuals. According to the consensus held by the International Continence Society (28), published in 2015, RT remains a major concern, and the greater likelihood of complications following AS implantation should be considered in this population.

Urethral atrophy can occur 4 years after AS placement and is likely a consequence of continuous urethral compression and poor vascular irrigation of the area, developing in 1.6% to 14% of cases (18, 22, 29, 30). In our series, 4.1 % of patients had urethral atrophy. When this condition occurs, the patient usually complains about the return of incontinence episodes. In our center, we prefer to implant a tandem cuff in these cases to improve continence status. Linder et al. (31) recently demonstrated that implantation of a second-cuff or downsizing of the cuff are both adequate procedures to treat the recurrence of the UI due to urethral atrophy.

The placement of the AS requires careful steps to achieve good device function. Malfunctions occurred in 1 individual (0.8%) in our study, which is a better result compared with other groups.

The perineal approach has improved outcomes versus scrotal access for AS placement. Henry et al. (32) reported that 56.5% of patients were completely dry after AS implantation through the classical perineal approach compared with 28.6% in the scrotal access group. In contrast to the Wilson's technique, we placed the cuff in the bulbous urethral and not in the penoscrotal transition. We use both perineal and scrotal access at our institution, although we prefer the former.

Scrotal implantation can be helpful, especially in patients with a previous perineal incision, such as after a sling procedure or removal of an AS. In our series, scrotal insertion was associated with more complications than the perineal approach, observing a 18.0% rate of erosion and extrusion with the scrotal technique versus 8.6% in the perineal group. This difference was not significant, likely due to the number of patients who were evaluated. Based on our concern over these data, we chose to use this approach in select cases, as discussed.

This a retrospective study and therefore it has some limitations, especially considering we did not use questionnaires and pad-test in our evaluation.

## CONCLUSIONS

This report is the largest single-center experience published in Brazil. Considering our outcomes, we conclude that AS implantation yields satisfactory results for the treatment of PPI and should remain the standard procedure for these patients. Radiation therapy is a risk factor for complications.
